# The necessary shift from diagnostic to prognostic research

**DOI:** 10.1186/1471-2296-8-53

**Published:** 2007-09-13

**Authors:** Geert-Jan GJ Dinant, Frank F Buntinx, Chris CC Butler

**Affiliations:** 1 Department of General Practice, School for Public Health and Primary Care, Maastricht University, Maastricht, the Netherlands; 2 Academic Centre for General Practice, Catholic University Leuven, Leuven, Belgium; 3 Department of General Practice, Cardiff University, Cardiff, UK

## Abstract

**Background:**

Do doctors really need to establish an etiological diagnosis each time a patient presents? Or might it often be more effective to treat simply on the basis of symptoms and signs alone, relying on research and on our experience of outcomes for patients who presented in similar ways in the past?

**Discussion:**

At a time of increase health care costs especially in pharmaceuticals and expensive diagnostic tests, this article uses examples from recent research to address this question. Our examples come from general practice, because that is where doctors frequently see patients presenting with a yet undifferentiated disease which is consequently difficult to diagnose. The examples include respiratory tract infections, low back pain and shoulder pain. Finally we discuss the 'something is wrong' feeling.

**Summary:**

We conclude that, in addition to diagnostic research, a renewed focus on prognostic research is needed.

## Background

Formulating an etiological diagnostic hypothesis is a fundamental part of almost every medical encounter, regardless of clinical setting and patient characteristics. Without an etiological, or biomedical, diagnosis, many would argue that treatment choice will not be rational and that patients cannot be given a clear idea of prognosis. In addition, the patient cannot easily be reassured and the doctor might be left with a troubling feeling of uncertainty. It is for that reason that medical students are extensively trained in how to make a diagnosis and to avoid, virtually at all costs, missing a serious disease. One consequence may be that, at a time of increase health care costs especially in pharmaceuticals and expensive diagnostic tests, doctors do more laboratory and radiological testing than strictly necessary. [1] Diagnostic syndromes, like the irritable bowel syndrome and the chronic fatigue syndrome, are no exception to this. By definition, these syndromes are lacking a biomedical background. However, in patients suspected of suffering from these syndromes, physicians tend to perform even more diagnostic testing, predominantly for excluding presumed biomedical explanations of the presented complaints.

But do we, as doctors, really need to establish an etiological diagnosis each time a patient presents? Or might it often be more effective to treat simply on the basis of symptoms and signs alone, relying on research and on our experience of outcomes for patients who presented in similar ways in the past? This article uses examples from recent research to address this question. We conclude that, in addition to diagnostic research, a renewed focus on prognostic research is needed. [2-4] Our examples come from general practice, because that is where doctors frequently see patients presenting with as yet undifferentiated disease which is consequently difficult to diagnose. Furthermore, in general practice, patients generally feel free to express their fear of having the first sign of a serious disease. Indeed, establishing patients explanatory models and exploring their ideas, fear and expectations about their illness is part of the formal clinical method of general practice. [5,6] Patients will often share with their doctor what they heard from their colleagues or family, or what they read on the internet, for example that a prolonged cough may be a first symptom of lung cancer, or that fatigue is a sign of leukaemia.

## Discussion

### Do we still need pneumonia as a diagnosis?

In daily general practice, cough is one of the 'top three' reasons patients consult. In most cases, the cough is caused by a common cold, sinusitis, acute bronchitis, or an exacerbation of asthma or COPD. When a lower respiratory tract infection (LRTI) seems likely, the doctor will wonder whether this is due to a bacterial infection. One important reason for such speculation is that only a bacterial LRTI is likely to respond to treatment with antibiotics. However, on the basis of history taking, physical examination, and even radiographs, it is practically impossible to distinguish a bacterial from a viral LRTI. [7,8] Possibly for this reason, many clinicians focus on trying to diagnose or exclude pneumonia (instead of a bacterial LRTI). However, even this approach is fraught, since many patients with bacterial pneumonia may recover without antibiotic or appropriate antibiotic treatment. [9]

These considerations led to the attempt to identify symptoms and signs that might predict a prolonged (longer that four weeks) duration of illness in patients presenting with a cough caused by LRTI. Those whose presenting features suggest they are at higher risk of a prolonged clinical course may gain considerable benefit from antibiotic treatment, and unnecessary antibiotic prescriptions might be avoided in the remaining much larger group of LRTI patients. [10] Co-morbidity, and asthma in particular, appears to predict a prolonged duration of illness in LRTI. We now need to answer the question whether these patients with community acquired LRTI and types of co-morbidity benefit from treatment with antibiotics. [11,12] Patients at the milder end of the disease spectrum will have limited benefit from antibiotics, even if they have a bacterial infection.

### Are low back pain and shoulder pain satisfactory diagnoses?

Musculoskeletal complaints compete with respiratory tract symptoms in the 'top three' of most frequently encountered problems in general practice. [13-15] Lower back pain (LBP) in particular, together with pain of the hip, knee and shoulder are common reasons to consult. For most episodes of LBP and shoulder pain, there is no clear relationship between the precise 'anatomical lesion' and treatment effectiveness (once, of course, only after a more serious, even if rare, condition has been excluded). For that reason, clinical guidelines advise general practitioners to adopt a 'wait and see' policy, including symptomatic treatment and lifestyle advices, for at least 2–6 weeks, rather than investigating the patient radiologically or refer to physiotherapy. [16-18]

However, a considerable number of patients re-consult within a few weeks. [19-21] Educational interventions aimed at reducing negative attitudes and beliefs, as well as cognitive-behavioural therapy, active physical treatment and graded exercise therapy, all reduce work absence, functional limitation and pain (including pain related fear), and promote earlier return to normal daily activities. This is especially true for patients with (chronic) LBP and shoulder pain. [22-25] Instead of focussing primarily on an etiological or precise anatomical diagnosis, it might make more sense to explore the determinants of chronic LBP and shoulder pain on which general practitioners can intervene. Research may be more helpful to clinicians if it attempts to answer questions such as this rather than say differentiating one form of non-sinister back or shoulder pain from another. [20,26]

### 'Something is wrong' as a determinant of diagnosis and prognosis

General practitioners are often unable to make a formal diagnosis for a patient, but are struck with the feeling that something serious may be wrong. Such feelings tend to give important information, and the general practitioners ignore these at their peril, even if it does not result in pointing to a specific disease. This general feeling has been tested. In a group of 320 patients contacting their general practitioner with chest pain, general practitioners' gut feelings successfully predicted serious cardiac or respiratory disease in 82% of the cases. [27] Also in detecting rare, but serious infections (meningitis, sepsis, pneumonia, etc.) within a large group of acutely ill children, this 'feeling' is suggested to be an important help. [28] When the chest pain study was repeated in the emergency department of a large university hospital, the discriminative power of 'something is wrong' was much lower. It is not clear if this resulted from the immediate application of technological tests in all patients in such department, where serious diseases are so much more prevalent, or from the relative lack of expertise in young residents that first examine the new arriving patients. [29]

The importance of the 'something is wrong' sign has yet only been studied in emergency situations. The impression exists, however, that it may also be important in diagnosing serious, but less acute diagnoses, such as malignancy or even diabetes, and thus play a prognostic rather than diagnostic role.

## Summary

### The key word is time

We are not suggesting that making a diagnosis can be dispensed with. This will always remain a corner stone of daily clinical practice. Our examples do, however, suggest that the needs of clinicians and patients are often not met by a classical approach of diagnostic research, in which signs, symptoms and test results are compared in a cross-sectional way with a 'gold standard' diagnosis. The so-called delayed type cross-sectional study design has been suggested as a more useful approach since it incorporates time for a possible diagnosis to either become clear or the symptoms to resolve. [30] But a diagnosis remains no more and no less than one part of a management process that starts when a patient feels the need to consult his or her doctor and ends in death or when the symptoms are resolved. We have attempted to illustrate that a specific etiological diagnosis is not necessarily always an obligatory or even important element in this process. It may well be that treatment choices will be better supported by evidence relating signs and symptoms (syndromes) to outcomes, rather than finding better ways of making a precise etiological or anatomical diagnosis. Identifying early and managing differently those at higher risk for an adverse outcome, while adopting a 'wait and see approach' for most patients may be the best way forward. This is not to say that a 'wait and see approach' means not offering symptomatic treatment and labelling the nature of the problem in an understandable way. Time, however, is the key word in this approach to diagnosis.

## Competing interests

The author(s) declare that they have no competing interests.

## Authors' contributions

GD set up the design of the article and prepared the manuscript. FB and CB prepared the manuscript.

## Pre-publication history

The pre-publication history for this paper can be accessed here:



## References

[B1] van der Weijden T, Van Bokhoven MA, Dinant GJ, Van Hasselt CM, Grol RPTM (2002). Understanding laboratory testing in diagnostic uncertainty: a qualitative study in general practice. Br J Gen Pract.

[B2] Glasziou P (2002). The importance of prognostic research. Aust Fam Physician.

[B3] Jones R (2006). Clinical research in primary care needs urgent boost [Letter]. BMJ.

[B4] Rothwell PM (2006). Medical academia is failing patients and clinicians. BMJ.

[B5] Brown JB, Stewart M, McWilliam CL (1999). Using patient-centered method to achieve excellence in care for women with breast cancer. Patient Educ Couns.

[B6] Kleinman A (2003). Introduction: common mental disorders, primary care, and the global mental health research agenda. Harv Rev Psychiatry.

[B7] Hopstaken RM, Stobberingh EE, Knottnerus JA, Muris JWM, Nelemans P, Rinkens PELM, Dinant GJ (2005). Clinical items not helpful in differentiating viral from bacterial lower respiratory tract infections in general practice. J Clin Epidemiol.

[B8] Hopstaken RM, Witbraad T, Van Engelshoven JMA, Dinant GJ (2004). Inter-observer variation in the interpretation of chest radiographs for pneumonia in community-acquired lower respiratory tract infections. Clin Radiol.

[B9] Hopstaken RM, Coenen S, Butler CC (2005). Treating patients not diagnoses: challenging assumptions underlying the investigation and management of LRTI in general practice. J Antimicrob Chemother.

[B10] Hopstaken RM, Butler CC, Muris JWM, Knottnerus JA, Kester ADM, Rinkens PELM, Dinant GJ (2005). Do clinical findings in lower respiratory tract infection help general practitioners prescribe antibiotics appropriately? An observational cohort study in general practice. Fam Pract.

[B11] Hak E, Bont J, Hoes AW, Verheij TJ (2005). Prognostic factors for serious morbidity and mortality from community-acquired lower respiratory tract infections among the elderly in primary care. Fam Pract.

[B12] Hopstaken RM, Coenen S, Butler CC, Nelemans P, Muris JWM, Rinkens PELM, Kester ADM, Dinant GJ (2006). Prognostic factors and clinical outcome in acute lower respiratory tract infections; a prospective study in general practice. Fam Pract Advance Access.

[B13] Andersson GB (1999). Epidemiological features of chronic low-back pain. Lancet.

[B14] Picavet HSJ, Schouten JSAG, Smit HA (1996). Prevalences and consequences of low back pain in the MORGEN-project 1993–1995.

[B15] de Bruijn CMC, De Bie RA, Geraets JJXR, Goossens MEJB, Köke A, Van den Heuvel WJA, Van der Heijden GJMG, Dinant GJ (2005). Evaluation of an education and activation programme to prevent chronic shoulder complaints: design of an RCT. BMC Family Practice.

[B16] Winters JC, De Jongh AC, Van der Windt DAWM, Jonquière M, De Winter AF, Van der Heijden GJMG, Sobel JS, Goudswaard AN (1999). NHG-Standaard Schouderklachten. [Dutch College of General Practitioners' practice guideline 'Shoulder complaints']. Huisarts Wet.

[B17] Chavannes AW, Mens JMA, Koes BW, Lubbers WJ, Ostelo RWJG, Spinnewijn WEM, Kolnaar BGM (2005). NHG-Standaard Aspecifieke lagerugpijn. [Dutch College of General Practitioners' practice guideline 'Low back pain']. Huisarts Wet.

[B18] Mens JMA, Chavannes AW, Koes BW, Lubbers WJ, Ostelo RWJG, Spinnewijn WEM, Kolnaar BGM (2005). NHG-Standaard Lumbosacraal radiculair syndroom. [Dutch College of General Practitioners' practice guideline 'Low back pain with radiation']. Huisarts Wet.

[B19] von Korff M, Saunders K (1996). The course of back pain in primary care. Spine.

[B20] Kuijpers T, Van der Windt DA, Van der Heijden GJ, Bouter LM (2004). Systematic review of prognostic cohort studies on shoulder disorders. Pain.

[B21] van der Windt DA, Koes BW, Boeke AJ, Deville W, Jong BA, Bouter LM (1996). Shoulder disorders in general practice: prognostic indicators of outcome. Br J Gen Pract.

[B22] Buchbinder R, Jolley D, Wyatt M (2001). Population based intervention to change back pain beliefs and disability: three part evaluation. BMJ.

[B23] Geraets JJXR, Goossens MEJB, de Groot IJM, de Bruijn CPC, De Bie RA, Dinant GJ, Van der Heijden GJMG, Van den Heuvel WJA (2005). Effectiveness of a graded exercise therapy program for patients with chronic shoulder complaints. Australian Journal of Physiotherapy.

[B24] Moore JE, Von Korff M, Cherkin D, Saunders K, Lorig K (2000). A randomized trial of a cognitive-behavioural program for enhancing back pain self care in a primary care setting. Pain.

[B25] Smeets REJM, Vlaeyen JWS, Hidding A, Kester ADM, Van der Heijden GJMG, Van Geel ACM, Knottnerus JA (2006). Active rehabilitation for chronic low back pain: Cognitive-behavioural, physical, or both? First direct post-treatment results from a randomized controlled trial. BMC Musculoskeletal Disorders.

[B26] Picavet HSJ, Schouten JSAG (2003). Musculoskeletal pain in the Netherlands: prevalences, consequences and risk groups, the DCM(3)-study. Pain.

[B27] Buntinx F, Truyen J, Embrechts P, Moreel G, Peeters R (1991). Chest pain: an evaluation of the initial diagnosis made by 25 Flemish general practitioners. Fam Pract.

[B28] van den Bruel A, Bartholomeeusen S, Aertgeerts B, Truyers C, Buntinx F (2006). Serious infection in children: an incidence study in family practice. BMC Family Practice.

[B29] Buntinx F, Knockaert D, De Blaey N, Aerts M, Bruyninckx R, Knottnerus JA, Delooz JA (2001). Chest pain in general practice or in the hospital emergency department: is it the same?. Fam Pract.

[B30] Knottnerus JA, Dinant GJ (1997). Medicine based evidence, a prerequisite for evidence based medicine. Br Med J.

